# From fields to cities: Innovating assessment of soil quality in Southern Iran’s Urban areas

**DOI:** 10.1371/journal.pone.0321312

**Published:** 2025-05-09

**Authors:** Sayyed Mahmoud Enjavinezhad, Seyed Javad Naghibi, Morteza Poozesh Shirazi, Majid Baghernejad, María Fernández-Raga, Jesús Rodrigo-Comino

**Affiliations:** 1 Department of Soil Sciences, School of Agriculture, Shiraz University, Shiraz, Iran; 2 Head of Research & Extension Office, Landscape & Green Spaces Organization of Shiraz Municipality, Shiraz, 45366-78, Iran; 3 Department of Soil and Water Research, Fars Agricultural and Natural Resources Research and Education Center, AREEO, Shiraz, Iran; 4 Department of Applied Physics and Chemistry, Industrial Engineering School, University of León, León, Spain; 5 Departamento de Análisis Geográfico Regional y Geografía Física, Facultad de Filosofía y Letras, Universidad de Granada, Granada, Spain; 6 Andalusian Research Institute in Data Science and Computational Intelligence (DaSCI), University of Granada, Granada, Spain; ICAR-National Rice Research Institute, INDIA

## Abstract

Evaluation of soil quality in urban and peri-urban areas using comparable and reproducible indexes is a necessary step to assess the soil management status and its potential for different uses. The application of quantitative indexes guarantees neutrality and reliability of results, allowing comparisons between areas with similar environmental soil conditions. However, there is no consensus on the application of specific indexes. Therefore, in this research, three indexes (Integrated, Weighted Integrated, and Nemoro´s quality indexes) and two approaches (linear and non-linear methods) were compared to select the most relevant soil properties for evaluating soil quality for different land uses (e.g., agriculture, gardening, parking, rangelands, or bare areas). To this end, an experimental area was selected with a total dataset of 25 physicochemical and biological properties in the Shiraz urban watershed (southern Iran). Nine soil properties were selected using the principal component analysis method as the most informative factors, forming the minimum dataset. The results showed that gardens and bare land had the highest (SQI = 0.34–0.55 across different approaches) and lowest soil quality index (SQI = 0.25–0.44 across different approaches), respectively. The non-linear index calculation approach had better efficiency than the linear one. According to the coefficients of determination (R^2^ = 0.81–0.89), these key soil variables were suggested as a solution to reduce both the cost and time required for projects carried out by experts and watershed decision-makers to assess soil quality in urban and peri-urban areas.

## 1. Introduction

Soils are vital for human life, as they support ecosystem biodiversity and functions. In addition, soils influence the quantity and quality of food and fiber production [1] and the way humans manage them determines the future of this resource [2,3]. The sustainable use of soil resources is essential for long-term human health and the conservation of quality of life [4,5]. It is well-known that soils are considered a non-renewable resource on timescales relevant to agricultural production and economic development due to their extremely slow formation after degradation [6].

Generally, soil quality (SQ) is defined as the capacity of soil to perform ecological functions and provide ecosystem services that sustain biological productivity, maintain environmental quality, and enhance plant and animal health [7,8]. In simpler terms, soil quality unifies physical, chemical, and biological components and processes along with their interactions [9], including functions such as sustaining plant and microbial productivity, regulating water and nutrient cycles, supporting biodiversity, mitigating environmental impacts, and maintaining ecosystem stability and resilience. Soil quality reflects the soil’s ability to adapt to natural and anthropogenic changes while ensuring long-term ecosystem health and productivity.

Thus, a comprehensive description of soil quality should rely on these multiple properties and functions [10], together with the soil`s capacity to function within the ecosystem and land use boundaries [11,12].

Particularly, peri-urban and urban soil formation is affected by both direct and indirect anthropogenic effects, which lead to distinct characteristics and processes [13]. Indirect anthropogenic effects on soil forming factors include changes in carbon and nitrogen stocks as well as soil temperature and moisture regimes [14]. According to the United Nations report in 2016, 54.5% of the world’s population lived in urban areas, and the number of urban residents is constantly increasing. Furthermore, 60% of the population is projected to live in urban areas by 2030, with a decrease in urban environmental quality. Therefore, appropriate public health and environmental assessments are essential [15]. Physical properties in soils classified as *Anthrosols* or *Tecnosols* [16], for instance, are affected by human activities such as over-compaction due to the use of heavy machinery, while chemical soil properties are also affected by processes like salinization, contamination, plastic depositions, and variations in soil reaction [17,18]. Furthermore, some factors such as parent materials, land use, and the type and intensity of anthropogenic pressure, control urban soil properties ultimately determine their quality and status [19].

For instance, Raiesi et al [20] introduced the minimum dataset and soil quality index to quantify the effect of land use conversion on soil quality and degradation in native rangelands of upland arid and semiarid regions. In this study, the soil organic carbon, electrical conductivity, and arylsulphatase activity were found to be the key indicators within the minimum dataset, significantly affecting soil quality. Since soil quality is a multifaceted functional concept and cannot be measured directly in-field or in the laboratory, it should be inferred from soil properties and processes related to land use and management [21].

These findings demonstrate the assessment of SQ allows the successful discrimination between rangeland and cropland ecosystems and quantifies the effects of land use conversion on SQ. Additionally, other researchers have reported that land use changes significantly affect soil quality when using both the Integrated Quality Indexing (IQI) and Cornell University methods [22].

For the selection of key indicators, all available information about the study area should be considered, including expert opinions and reviews of previous studies [23]. The Soil Quality Index (SQI) integrates critical indicators and simplifies of complex information by quantifying and communicating the most relevant soil quality properties to support optimal soil management decisions [24,25]. The use of minimum datasets in determining soil quality provides the most economical and reliable results while ensuring data quality [26]. Various mathematical and statistical methods are used to calculate SQI [27]. For example, principal component analysis (PCA) using basic components identifies common effects of multiple variables. By applying PCA, the dependency structure among variables is eliminated [26].

Data normalization is a necessary process when scoring the indicators due to the different numerical scales. Among all the data normalization techniques, one of the most commonly used for its simplicity is chart rating, which initially rates indicators based on measured values and then assigns scores to each rating [28]. Another normalization method is linear scoring (LS), which establishes a linear relationship between the quality score and measured data, depending on the sensitivity of the indicator to changes in the soil quality [29]. Additionally, the non-linear scoring (NLS), method which depends on measured values of indicators, can be used when there is no linear relationship between quality scores and indicator values [23].

To understand the difference between the scoring methods, considering soil organic carbon (SOC) and soil reaction as two indicators is recommended. Generally, higher SOC is associated with better soil quality, and a linear relationship can be observed between SOC and its quality score. If SOC ≤ 1%, the soil quality score equals 0, and if SOC ≥ 3%, the soil quality score equals 1. A linear equation is used to interpolate the quality score for SOC values between 1% and 3% (e.g., Quality Score = (SOC - 1)/2) [29]. On the other hand, soil with neutral pH (around 6.5 to 7.5) is typically associated with optimal soil quality. As values fall below or rise above this range, soil quality decreases. Therefore, there is a non-linear relationship between soil reaction and its quality score. If pH = 7.0, the soil quality score = 1, and if pH ≤ 5.0 or pH ≥ 9.0, the soil quality score = 0. Thus, a non-linear scoring curve can reflect a decrease in quality as pH deviates from 7.0 [23].

As mentioned above, the selection of a specific method to assign indicator scores or values to each indicator is crucial because it is the combination of scores from selected indicators that forms an SQI, which can be produced through several systematic approaches. Calculation methods such as averaging, summing, and multiplying are simple to use but do not account for differences in the contribution of each indicator to soil quality [30,31].

There are two main methods to calculate the SQI: Integrated Quality Index (IQI) and the Nemoro Quality Index (NQI). The IQI method considers the importance of each indicator and assigns a weight value to each indicator during score indexing based on expert opinion or statistical analysis [32]. While the NQI method focuses on the impact of limiting factors on soil quality, using minimum and average indicator scores [33]. Both quality indexing methods (i.e., IQI and NQI) and their different scoring methods are widely applied as valuable tools for assessing the impact of land use on soil quality in agricultural soils [34, 35, 36] and vegetation restoration [37,38]. However, soil quality in urban and peri-urban areas is often assessed using other indexing methods, including Soil Biological Quality (QBS-ar) index [39], soil enzyme-based index [40], soil evaluation factor [41], structural soil quality index [42], and soil SOM quality index [43], among others.

Generally, such studies have been conducted in at least one land use category within urban and peri-urban areas such as grasslands, agricultural degraded vacant lots, parks, and gardens. Therefore, a comparison between different land uses within these areas is necessary due to the constant influence of urbanization. Only a few studies have applied IQI and NQI indexing methods to a single specific land use in urban and pre-urban areas or to reclaimed land in the urban–rural fringe [44,45]. Thus, it is vital to assess the effectiveness of these two soil quality indexing methods and their different scoring methods across various land uses of urban and peri-urban areas.

Not only does the method define the final result, but also the tool used to for mapping. Geographic information systems (GIS) are one of the most powerful modern monitoring techniques for evaluating SQI [46]. However, GIS-based studies related to SQI evaluation methods in urban and peri-urban are not common. The referenced data can be manipulated, stored, and updated in GIS to support urban planning and decision-making such as determining the optimal location for infrastructure or recommending specific land uses. These datasets can be integrating into GIS at the regional or national level incorporated into spatial modeling tools [47]. For example, a GIS-based model was developed to assess SQI in the Northeast Nile Delta by rating, weighting, and overlaying thematic layers of chemical indicators [48]. However, the factors affecting urban soil quality involve many variables that are highly sensitive to slight changes in land use. As a result, few studies have compared and identified the most appropriate SQI determination method for assessing the urban soil quality.

Therefore, this study aimed to determine the minimum effective dataset for SQI assessment in urban and peri-urban soils, using a degraded area in Shiraz (Iran) as a case study. Based on this information, a comparison of soil quality evaluation was performed using the IQI and NQI methods, applying both linear and non-linear scoring methods influenced by land use. Additionally, to enhance the comparison, each SQI evaluation method was mapped using GIS.

## 2. Methods and materials

### 2.1. Study area

**Fig 1:**
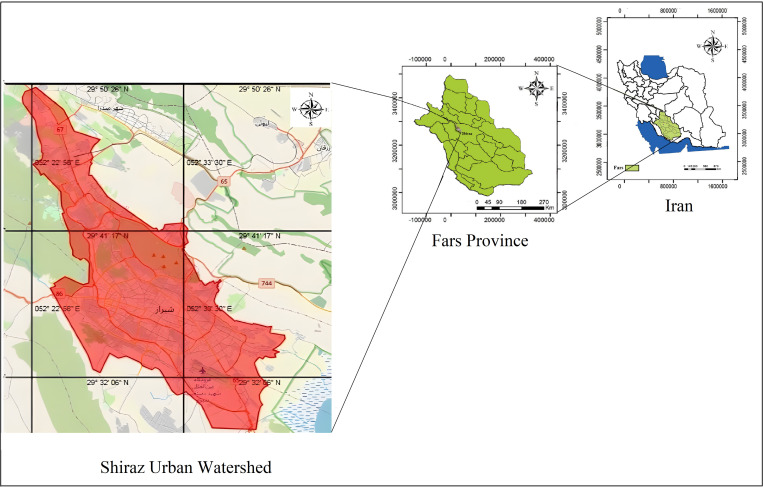
Study area (the original maps were obtained from https://earthexplorer.usgs.gov and modified as necessary by the authors).

The current study was conducted in the Shiraz urban watershed, located in Fars province, southern Iran, covering an area of 411,133 hectares. The experimental area is situated between the latitudes 662390 and 632643 north and longitudes 3262310 and 3301466 east, in the 39R zone (metric coordinate system; Fig 1). The main drainage of this watershed consist of the Khoshk and Mianrud rivers, and the basin´s outlet is Maharlu Lake, located southeast of Shiraz, which is actually a playa. The soil moisture and temperature regimes are xeric and thermic, respectively.

The Shiraz watershed is a part of the Folded Zagros Sedimentary Basin, with folds oriented roughly northwest-southeast in alignment with this zone. From a stratigraphic perspective, this are including a sequence of different rock facies, ranging from the Miocene period to the present. In the experimental area, there are no outcrops from pre-Miocene periods, and deposits from later periods have been systematically layered on top of each other. The lithology of the surrounding heights is mainly composed of limestone and dolomitic limestone [49].

**Fig 2:**
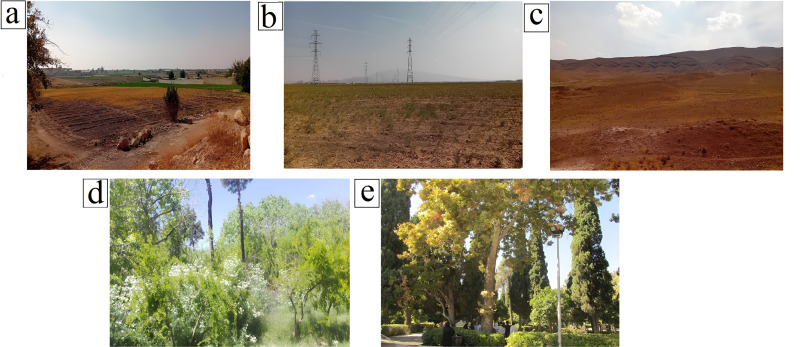
Different land uses in the Shiraz urban watershed: (a) Agriculture, (b) Bare land, (c) Rangeland, and (d) Garden, (e) Urban park. (Photos taken by the authors).

To evaluate soil quality in the Shiraz urban watershed, four different land uses were investigated including bare lands, rangelands, gardens, and urban parks (Fig 2). The native flora species in the Shiraz watershed include *Capparisspinosa parviflora*, *Dianthus crinitus*, *Achillea eriophora*, *Artemisia herbaalba*, *Centaurea bruguierana*, *Convolvulus argyrothamnus, Avena fatua*, *Hordeum spontaneum*, *Papaver rhoeas*, *Ficus carica*, *Pistacia atlantica*, *Amygdalus eleagnifolia*, *Amygdalus scoparia*, and *Cerasus microcarpa*. The common garden flora species in the experimental area are *Vitis vinifera, Punica granatum*, *Prunus dulcis*, *Diospyros kaki*, and *Citrus × aurantium*; while the major flora in urban parks included *Platanus spp*, *Acer spp, Ficus religiosa*, *Cupressus sempervirens*, *Rosa damascene*, and *Zinnia elegans* [50].

### 2.2. Soil sampling

A total of 150 topsoil samples were extracted from depth of 0–20 cm [51]. Samples were obtained using a systematic random method with a Garmin 62s GPS device, ensuring coverage of different land uses, including bare lands, rangelands, agricultural areas, gardens, and urban parks (Fig 2). After being transported to the laboratory, the soil samples were air-dried and sieved through a 2 mm mesh for further analysis.

### 2.3. Soil quality indicators measurement

A literature review was conducted to select the variables and measurement methods for soil properties (physical, chemical, and biological) used in the initial dataset for evaluating the Soil Quality Index (Table 1). Based on previous studies and available laboratory facilities, a total of five physical properties, 16 chemical properties, and four biological properties were identified as indicators affecting soil quality. These indicators constitute the Total Data Set (TDS) for this study [52].

**Table 1:** Soil properties involved in a dataset for evaluation of soil quality index.

Acronyms		Physical properties	
Sand, Silt, Clay	Texture		[53,54]
BD	Bulk density (g cm^-3^)		[55]
MWD	Mean weight diameter (mm)		[56]
AW	Available water (cm)		[57]
SP	Saturation percentage (%)		[58]
**Chemical properties**			
pH	pH		[59]
EC	Electrical conductivity (dS m^-1^)		[59]
TDS	Total dissolved salt (mg kg^-1^)		[60]
TNV	Total neutralizing value (%)		[61]
OC	Organic carbon (%)		[62]
CEC	Cation exchange capacity (cmol(+) kg^-1^)		[63]
N	Total nitrogen (%)		[64]
P	Available phosphorus (mg kg^-1^)		[65]
K	Available potassium (mg kg^-1^)		[59]
Pb	Lead (mg kg^-1^)		[66]
Cd	Cadmium (mg kg^-1^)		[66]
As	Arsenic (mg kg^-1^)		[66]
Cr	Chromium (mg kg^-1^)		[66]
Ni	Nickel (mg kg^-1^)		[66]
Co	Cobalt (mg kg^-1^)		[66]
Fe	Iron (mg kg^-1^)		[66]
**Biological properties**			
M-Res	Microbial respiration (mg CO2-C kg^-1^ soil h^-1^)		[67]
Bio-C	Biomass carbon (mg C kg^-1^ soil)		[68]
Bio-P	Biomass phosphorus (mg P kg^-1^ soil)		[68]
Bio-N	Biomass nitrogen (mg N kg^-1^ soil)		[68]

#### 2.3.1. Selection, weighting, and scoring of indicators.

The most important aspect in soil quality (SQ) assessment is the selection of optimal indicators that accurately reflect the state of soil quality status. These indicators should cover a wide range of soil properties and must have a direct and simultaneous impact on soil quality. In the present study, the total dataset included 25 biological, chemical, and physical soil properties (Table 1). Among these indicators, the minimum dataset of factors affecting soil quality was determined using principal component analysis (PCA).

Due to the variety of measurement units, initially, the indicators were first converted into unitless values for mathematical calculations (Table 2), through data standardization or ranking using standard scoring functions.

**Table 2:** Linear scoring functions for effective soil quality indicators.

Soil properties	Type of scoring function	L	U	O
Clay	More is better	10.74	37.52	
Silt	Less is better	18.90	54.10	
Sand	Less is better	35.20	75.68	
BD	Less is better	1.2	1.73	
MWD	More is better	0.62	4.04	
AW	More is better	8.29	19.28	
SP	Optimal range	19	60	45
pH	Optimal range	6.35	9.55	7
EC	Less is better	0.2	2	
TNV	Less is better	31.15	85.82	
CEC	More is better	2.34	17.97	
OC	More is better	0.35	4.87	
N	More is better	0.06	0.48	
P	More is better	1	72.1	
K	More is better	106	603	
Cr	Less is better	18.10	59.10	
Fe	Less is better	400	21700	
Co	Less is better	9.70	27.20	
Ni	Less is better	0.96	40	
As	Less is better	0.22	8.40	
Cd	Less is better	0.83	2	
Pb	Less is better	0.26	37.4	
M-Res	More is better	0.1	0.42	
Bio-C	More is better	222.62	3378.21	
Bio-P	More is better	10.05	43.17	

One indicator was qualitative and was categorized into three classes using the linear scoring (LS) functions [69], where L, U, and O represent the lower limit, upper limit, and optimal limit, respectively; and x the measured property (Table 2). M_(x)_ represents the “more-is-better” linear scoring function.

Depending on the behavior of the property, the equation follows one of three possible models:

1) A higher value of this property indicates better soil quality, following the “more-is-better” function (Equation 1). Soil organic carbon is a key example of this function;

$$if\;x\leq L\to M_{\left(x\right)}=0$$

$$if\;L\leq x\leq U\;\to M_{\left(x\right)}=\frac{x-L}{U-L}$$

$$if\;x\geq U\;\to M_{\left(x\right)}=1$$ (1)

2) A property where an increase leads to a decline in soil quality, following the “less-is-better” function (L_(x)_), is modeled by Equation 2. This applies to properties such as bulk density;

$$if\;x\leq L\to L_{\left(x\right)}=1$$

$$if\;L\leq x\leq U\;\to L_{\left(x\right)}=1-\frac{x-L}{U-L}$$

$$if\;x\geq U\;\to L_{\left(x\right)}=0$$ (2)

3) A property that has an optimum value (x), where any increase or decrease beyond this threshold reduces soil quality, follows the “optimal range” function (OR_(x)_) (Equations 3a and 3b). This applies to properties such as soil reaction (pH), where deviations from the optimal range negatively affect soil quality.

$$if\;x<0\;\&\;x\leq L\to {OR}_{\left(x\right)}=0$$

$$if\;x<0\;\&\;L\leq x\leq U\;\to {OR}_{\left(x\right)}=\frac{x-L}{U-L}$$

$$if\;x<0\;\&\;x\geq U\;\to {OR}_{\left(x\right)}=1$$ (3a)

$$if\;x>0\;\&\;x\leq L\to {OR}_{\left(x\right)}=1$$

$$if\;x>0\;\&\;L\leq x\leq U\;\to {OR}_{\left(x\right)}=1-\frac{x-L}{U-L}$$

$$if\;x>0\;\&\;x\geq U\;\to {OR}_{\left(x\right)}=0$$ (3b)

Another approach used for data standardization or ranking is the non-linear scoring function (NLS) [70], which follows Equation 4.


$$NLS=\frac{1}{1+{\left(\frac{x}{x_{m}}\right)}^{b}}$$ (4)

Where NLS is the non-linear scoring function, *X* represents the measured soil property, *X*_m_ is the mean of the desired property, and *b* is the slope of the equation, which is + 2.5 for the “more-is-better” equation and -2.5 for the “less-is-better” equation.

#### 2.3.2. Soil quality indexes.

Soil quality in arid and semi-arid regions has been calculated using three indexes: Integrated (Equation 5), Weighted Integrated (Equation 6), and Nemoro (Equation 7). The Integrated Soil Quality Index is a summation of allocated scores obtained from minimum dataset [71]. The Weighted Integrated Soil Quality Indexing method considers the importance of each indicator, then determines the weight value of each indicator through the indexing of scores [32,72]. The allocation of weight values is done according to expert opinion or statistical analysis [28,73], while Nemoro Quality Indexing method considers the minimum and average indicator scores in the assessment process [74].


$${SQI}_{a}=\frac{\Sigma_{i}^{n}N_{i}}{n}$$ (5)

Where *SQIa* is the Cumulative Index of Soil Quality, *Ni* is the score of soil characteristics, and *n* is the number of soil properties [75].


$${SQI}_{w}=\Sigma_{i=1}^{n}W_{i}*S_{i}$$ (6)

Where *Si* is the score of the indicator, *n* is the number of indicators, and *Wi* is the weight of each indicator. The weight value was assigned to each parameter based on the commonality of each indicator, calculated factor analysis (SPSS v.26, IBM). The weight value for each parameter was determined as a ratio of its commonality relative to the overall commonality [76].


$$NQI=\sqrt{\frac{P_{ava}^{2}-P_{\min}^{2}}{2}}*\frac{n-1}{n}$$ (7)

Where *P*_*av*a_ is the average score awarded to selected properties in each soil sample, *P*_*min*_ is the lowest score among the selected properties for each soil sample, and n is the number of soil properties [72].

#### 2.3.3 Soil quality mapping.

Soil quality mapping was implemented by ArcGIS 10.5 (ESRI, USA) software, applying the kriging estimator method and inverse distance weighting (IDW) for each soil quality index. To determine the best interpolation method for estimating the soil quality maps, the lowest root mean square error and absolute mean error were used (Table 3).

**Table 3:** Selected models for mapping soil quality indicators using different interpolation methods.

	IDW		Kriging		Chosen model
	**RMSE**	**MAE**	**RMSE**	**MAE**	
SQIw-TDS-L	0.09	0.005	0.08	–0.001	Kriging
SQIw-TDS-N	0.08	0.005	0.08	–0.001	Kriging
SQIw-MDS-L	0.12	0.006	0.11	–0.001	Kriging
SQIw-MDS-L	0.11	0.005	0.1	–0.009	Kriging
SQIa-TDS-L	0.08	0.004	0.08	–0.001	Kriging
SQIa-TDS-N	0.08	0.004	0.07	–0.005	Kriging
SQIa-MDS-L	0.11	0.006	0.11	–0.001	Kriging
SQIa-MDS-N	0.10	0.005	0.98	–0.008	Kriging
SQIn-TDS-L	0.06	0.003	0.06	–0.001	Kriging
SQIn-TDS-N	0.07	0.003	0.07	0.0002	Kriging
SQIn-MDS-L	0.08	0.004	0.07	–0.001	Kriging
SQIn-MDS-N	0.1	0.005	0.09	0.001	Kriging

RMSE: Root Mean Square Error MAE: Mean Absolute Error

Note: The names are composed by three parts, describing the method followed (a-b-c):

a) Soil quality index used: SQIw (Weighted Integrated), SQIa (Integrated); SQIn (Nemoro).

b) Dataset analysis: TDS (for total dataset) or MDS (for minimum dataset).

c) Two options for approach: L (linear approach) or N (nonlinear approach).

Note: This study was conducted in Shiraz urban watershed, Fars Province, southern Iran, which is one of the study areas examined by Research and Extension Office, Landscape and Green Spaces Organization of Shiraz Municipality, IR Iran. It should be noted that the second author is a member of academic staff of Research and Extension Office, Landscape and Green Spaces Organization of Shiraz Municipality and no special permission was required to access the field site.

## 3. Results and discussions

### 3.1. Descriptive statistics

The descriptive statistics of the total dataset in Shiraz urban soils are shown (Table 4), where the average soil texture in the study area was loam, but with the predominant effect of sand (44.31%), and silt (38.21%) particles are more represented, with clay less present (17.47%). Some other loam-based textures were found in the area with a variability of less than 0.35. The soil bulk density showed low variability (coefficient of variation = 0.03), due to the entry and exit of soil from the urban environment, which modifies the urban cultivation beds and also leads to soil compaction, which modifies the urban cultivation beds and also leads to soil compaction, resulting in uniform changes in the urban environment. Additionally, the MWD variability (CV = 0.26) in the area can support this observation. Properties such as EC and pH indicated that the soils of the study area were neutral to relatively alkaline, with no sign of salinity problem, except for samples taken near Maharlu Lake.

**Table 4:** Descriptive statistics.

Soil properties	Units	Minimum	Maximum	Mean	Standard deviation	coefficient of variation	Skewness	Kurtosis	Kolmogorov-Smirnov test
Clay	(%)	7.6	33.5	17.47	5.51	0.32	**0.44**	**-0.59**	0.003
Silt	(%)	18.90	54.10	38.21	5.58	0.15	**-0.48**	**0.87**	0.478
Sand	(%)	21.90	72.60	44.31	10.06	0.23	**0.13**	**-0.11**	**0.566**
BD	(g cm^-3^)	1.28	1.51	1.40	0.05	0.03	**0.40**	**0.84**	0.000
MWD	(mm)	0.62	4.04	3.05	0.81	0.26	**-1.29**	**0.74**	0.000
AW	(cm)	11.11	18.82	14.28	1.49	0.10	**0.42**	**1.14**	0.016
SP	(%)	23.00	48.00	33.03	5.67	0.17	**0.58**	**-0.45**	0.003
pH	–	7.02	8.77	7.69	0.21	0.03	**0.49**	3.75	**0.581**
EC	(dS m^-1^)	0.41	16.32	2.74	2.02	0.74	3.02	14.39	0.001
TNV	(%)	31.15	85.82	52.68	7.19	0.14	**0.47**	2.59	0.183
CEC	(cmol(+) kg^-1^)	4.58	20.91	12.32	3.87	0.31	**0.01**	**-0.69**	**0.636**
OC	(%)	0.01	3.90	1.79	1.23	0.69	**0.01**	**-1.47**	0.028
N	(%)	0.00	0.41	0.19	0.13	0.65	**-0.13**	**-1.37**	0.029
P	(mg kg^-1^)	0.15	20.10	6.54	4.31	0.66	**0.66**	**0.32**	0.385
K	(mg kg^-1^)	106.00	603.00	315.00	84.89	0.27	**0.01**	**0.04**	**0.658**
Cr	(mg kg^-1^)	0.00	167.00	89.53	31.22	0.35	**-0.42**	**0.54**	0.175
Fe	(mg kg^-1^)	400.00	21700.00	8441.90	2608.44	0.31	**0.51**	5.55	0.053
Co	(mg kg^-1^)	0.40	25.30	12.54	4.49	0.36	**-0.20**	**0.23**	0.455
Ni	(mg kg^-1^)	0.96	218.10	121.77	30.96	0.25	**-0.64**	3.18	0.200
As	(mg kg^-1^)	0.22	8.40	0.52	0.72	1.39	9.34	98.51	0.000
Cd	(mg kg^-1^)	0.83	7.93	2.07	0.62	0.30	5.55	54.91	0.000
Pb	(mg kg^-1^)	5.12	17.40	7.78	1.22	0.16	3.32	25.23	0.053
M-Res	(mg CO2-C kg^-1^ soil h^-1^)	0.10	0.42	0.26	0.09	0.37	**0.22**	**-1.18**	0.005
Bio-C	(mg C kg^-1^ soil)	222.62	3378.21	1770.00	968.23	0.55	**0.15**	**-1.00**	0.046
Bio-P	(mg P kg^-1^ soil)	10.05	43.17	25.45	11.04	0.43	**0.03**	**-1.47**	0.000

The ranges for evaluation will be

For coefficient of variation three ranges of differences: Very low (CV < 0.1), Low (0.1 < CV < 0.2), Medium (0.2 < CV < 0.3), high (CV > 0.3).

For Skewness and Kurtosis values normal are those in between -2 and 2.

In Kolmogorov Simonov test the signification is for values higher than 0.05.

The meaning of some symbols: BD (Bulk density); MWD (Mean weight diameter); AW (Available water); SP (Saturation percentage); EC (Electrical conductivity); TNV (Total neutralizing value); CEC (Cation exchange capacity); OC (Organic carbon); M-Res (Microbial respiration); Bio-C (Biomass carbon); Bio-P (Biomass phosphorus).

According to the TNV percentage, soils in the study area were calcareous [77]. The soils also registered low to moderate levels of organic carbon (1.79%), due to the influence of the semi-arid climate conditions and the lack of sufficient moisture for the annual organic substances to the soil system. The variability of carbon content was high (CV = 0.69) due to different land uses in urban areas. The cation exchange capacity was low (12.32 meq/100g), which was in line with the variability of organic matter and clay. The other chemical and biological properties showed medium (CV of Pb = 0.16) to high variability (CV of As = 1.39), which might be due to anthropogenic changes (i.e., fertilizer application, pollution, etc.) in urban areas.

### 3.2. Minimum dataset affected SQ

To select the minimum dataset (MDS), the feature with the highest weight along with those that differed by less than 10% from it, was chosen as the set of minimum features affecting soil quality (Table 5). If more than one feature was selected for each component, those with high correlation coefficients (r > 0.6) and lower weights were removed [75].

**Table 5:** Minimum dataset selection.

	Principle Components							Total dataset		Minimum dataset	
	**PC1**	**PC2**	**PC3**	**PC4**	**PC5**	**PC6**	**PC7**	**Communalities**	**Weight**	**Communalities**	**Weight**
**Eigenvalue**	2.88	2.78	2.43	1.93	1.50	1.24	1.01				
**Percentage of variance**	11.53	11.10	10.91	7.70	6.41	6.16	5.67				
**Cumulative percentage**	11.53	22.63	33.54	41.24	47.64	53.81	59.48				
**Eigenvectors**											
**Clay**	0.01	0.64	0.24	-0.08	0.31	0.18	-0.15	0.68	0.04		
**Silt**	-0.02	**0.87**	0.05	0.16	-0.07	-0.01	-0.19	0.84	0.05		
**Sand**	-0.06	**-0.95**	-0.07	-0.01	0.05	0.02	0.18	0.95	0.05	0.63	0.11
**BD**	-0.13	0.08	-0.06	**0.85**	0.12	0.07	0.03	0.78	0.04	0.39	0.07
**MWD**	0.71	0.26	0.33	-0.17	0.15	0.12	0.14	0.79	0.04		
**AW**	-0.07	0.13	-0.06	**0.83**	0.02	0.001	0.01	0.73	0.04		
**SP**	0.06	0.61	-0.28	0.33	0.04	-0.16	0.25	0.66	0.04		
**pH**	0.16	-0.05	0.06	0.02	-0.06	0.29	-0.03	0.70	0.04		
**EC**	0.13	-0.21	-0.15	0.06	-0.07	-0.03	**0.75**	0.67	0.04	0.64	0.11
**TNV**	-0.42	-0.15	0.01	-0.04	-0.13	0.05	0.61	0.62	0.03		
**CEC**	0.08	0.004	-0.05	0.09	-0.13	**0.76**	-0.15	0.65	0.04	0.72	0.13
**OC**	**0.89**	-0.04	0.29	-0.06	0.08	0.03	-0.06	0.90	0.05		
**N**	**0.98**	-0.05	0.29	-0.06	0.07	0.04	-0.07	0.92	0.05	0.68	0.12
**P**	0.20	-0.20	0.11	0.36	-0.02	-0.07	-0.20	0.69	0.04		
**K**	0.01	-0.004	-0.06	-0.04	0.13	**0.75**	0.14	0.62	0.03	0.68	0.12
**Cr**	0.08	-0.03	0.14	0.08	**0.83**	-0.02	0.04	0.75	0.04	0.66	0.12
**Fe**	0.13	0.05	-0.08	0.04	**0.74**	0.02	-0.17	0.64	0.03	0.64	0.11
**Co**	0.10	-0.15	0.10	0.03	-0.02	0.22	-0.06	0.66	0.04		
**Ni**	0.10	0.12	-0.11	0.31	0.36	-0.19	-0.29	0.60	0.03		
**As**	-0.05	0.13	-0.05	-0.24	0.03	0.01	0.12	0.75	0.04		
**Cd**	0.38	-0.05	-0.31	0.06	0.09	-0.33	-0.25	0.66	0.04		
**Pb**	-0.27	0.02	-0.05	0.12	0.06	-0.17	-0.05	0.72	0.04		
**M-Res**	0.3	0.13	**0.82**	-0.11	0.08	-0.02	0.05	0.84	0.05		
**Bio-C**	0.33	0.17	**0.85**	-0.08	0.06	-0.03	-0.14	0.89	0.05		
**Bio-P**	0.18	-0.14	**0.86**	0.02	-0.05	-0.09	-0.08	0.82	0.04	0.61	0.12

* Bold numbers were considered the most weighted (10%). Underlined bold numbers were selected as MDS.

**Fig 3:**
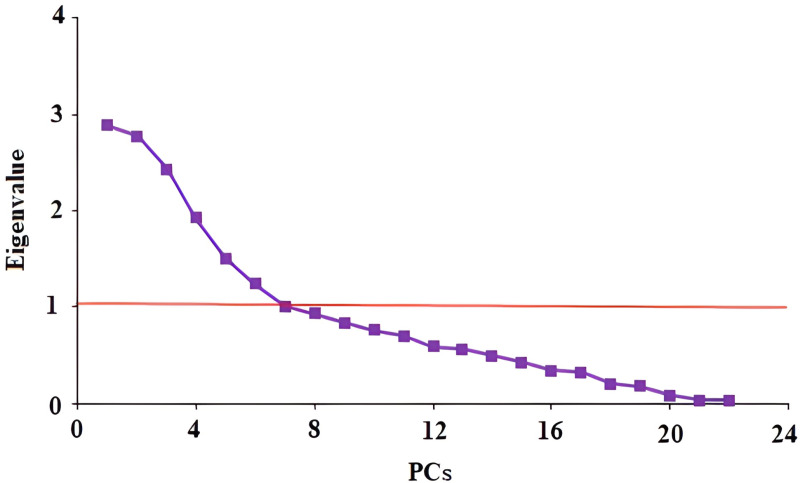
Scree plot of PCs.

The results of PCA showed that seven principal components (PCs) had eigenvalues greater than 1, which together explained more than 59% of the total variance. In other words, each of these seven PCs explained 11.53, 22.63, 33.54, 41.24, 47.64, 53.81, and 59.48%, respectively (Table 5). To plot the components on the X-axis and the related eigenvalues on the Y-axis, the Cattel scree test [78] was performed (Fig 3). This plot always displays a downward curve. The point where the slope of the curve levels off (the “elbow”) indicates the number of factors that should be retained by the analysis [79]. In the scree plot, component extraction continues until the amount of specific variance is less than the common variance, i.e., before the specific variance surpasses the common variance. In other words, it continues until the share of common variance exceeds the share of specific variance [80].

Armenise et al. [81] stated that if the correlated variables in a component are important factors affecting soil quality, they should remain in the MDS due to their importance. Therefore, to find correlated variables in each PC, Pearson correlation results were used (Table 6). In PC1, nitrogen content (N) had a significant positive correlation (P < 0.01) with organic carbon content (OC), which could serve as an indicator for an agronomic goal since this nutrient most often limits crop production [79]. In PC2, silt particles (Silt) had a negative correlation (P < 0.01) with sand particles (Sand), while showing significant positive correlations (P < 0.05) with available water (AW) and bulk density (BD). Also, sand particles showed negative correlations with microbial respiration (M-Res) (P < 0.05) and biological carbon (Bio-C) (P < 0.01), respectively. Thus, the selection of sand particles in MDS might be due to their importance in influencing biological properties. The reason for such a finding could be the highly modifiable nature of soil physical properties on a small scale, especially in urban areas. In PC3, all three most weighted eigenvectors had positive correlations with each other. Thus, biological phosphorus (Bio-P) was selected in MDS due to its ability to encompass interstitial effects. In PC4, bulk density and available water had a significant positive correlation (P < 0.05). Bulk density was selected due to its controlling effect on several soil properties (i.e., organic matter content, available water, infiltration rate, and pores volume). Also, soil compaction resulting from changes in bulk density, which is common in urban development and infrastructure maintenance, might be another reason to choose this property.

**Table 6:** Pearson’s correlation coefficient of components with the highest weight.

	Silt	Sand	BD	AW	CEC	OC	N	K	Cr	Fe	M-Res	Bio-C	Bio-P
**Silt**	1												
**Sand**	-0.91^**^	1											
**BD**	0.21^*^	-0.04	1										
**AW**	0.20^*^	-0.10	0.64^**^	1									
**CEC**	-0.001	-0.02	0.04	-0.002	1								
**OC**	-0.03	-0.04	-0.13	-0.14	0.09	1							
**N**	-0.03	-0.03	-0.14	-0.12	0.07	0.96^**^	1						
**K**	-0.01	0.04	0.03	-0.04	0.36^**^	-0.01	-0.01	1					
**Cr**	-0.02	0.03	0.10	0.03	-0.10	0.17^*^	0.17^*^	0.06	1				
**Fe**	0.04	-0.05	0.14	0.07	-0.02	0.14	0.14	0.02	0.37^**^	1			
**M-Res**	0.11	-0. 16^*^	-0.13	-0.13	-0.03	0.49^**^	0.48^**^	0.02	0.14	0.04	1		
**Bio-C**	0.15	-0.27^**^	-0.18^*^	-0.14	0.02	0.50^**^	0.51^**^	-0.03	0.12	0.08	0.82^**^	1	
**Bio-P**	-0.03	0.03	-0.11	-0.11	-0.06	0.38^**^	0.38^**^	-0.02	0.08	-0.05	0.66^**^	0.78^**^	1

* and

** are significant at the probability level of 1 and 5 percent, respectively.

In PC5, chromium (Cr), and iron (Fe) were added to MDS due to pollution concerns in urban areas and the iron deficiency commonly found in calcareous soils. In PC6, cation exchangeable capacity (CEC) and potassium content (K) were chosen for MDS due to their importance in plant productivity. Electrical conductivity (EC) selection for MDS in PC7 might be due to its influence on nutrient availability and salinity issues. In general, physical soil properties are affected by human activities such as over-compaction, while chemical soil properties are influenced by salinization and soil reaction variability [19]. Therefore, the current study suggests that MDS should be chosen to include all key processes and aspects involved in SQI.

### 3.3. The relationship between soil quality indexes and land use

Integrated, Weighted Integrated, and Nemoro indexes for Total Dataset (TDS) and Minimum Dataset (MDS) were determined for each land use (Table 7). Garden and bare land showed the highest and lowest mean values, respectively, in all indexes of both TDS and MDS. The comparison of indexes calculated with a linear approach between TDS and MDS showed that TDS was more accurate than MDS, as it contained more properties in the index calculation. This result contrasts with indexes calculated with indexes calculated using non-linear approach, which behaved differently, similar to the findings of Gorji et al. [52].

**Table 7:** Mean comparison of soil quality indices in different land uses.

Pr > F	Bare land	Rangeland	Agriculture	Park	Garden	SQI
**<0.0001**	0.41^c^	0.47^b^	0.46^b^	0.49^ab^	0.53^a^	**SQIw-TDS-L**
**<0.0001**	0.41^d^	0.46^bc^	0.45^c^	0.48^ab^	0.51^a^	**SQIa-TDS-L**
**<0.0001**	0.29^c^	0.32^bc^	0.31^c^	0.33^ab^	0.36^a^	**SQIn-TDS-L**
**<0.0001**	0.43^c^	0.49^b^	0.49^b^	0.52^ab^	0.54^a^	**SQIw-TDS-N**
**<0.0001**	0.44^c^	0.49^b^	0.49^b^	0.52^ab^	0.54^a^	**SQIa-TDS-N**
**<0.0001**	0.31^c^	0.36^b^	0.37^ab^	0.38^ab^	0.40^a^	**SQIn-TDS-N**
**0.0005**	0.37^c^	0.43^b^	0.43^b^	0.49^a^	0.50^a^	**SQIw-MDS-L**
**<0.0001**	0.38^c^	0.44^b^	0.43^b^	0.50^a^	0.51^a^	**SQIa-MDS-L**
**<0.0001**	0.25^c^	0.29^b^	0.29^b^	0.33^a^	0.34^a^	**SQIn-MDS-L**
**<0.0001**	0.43^c^	0.49^bc^	0.49^bc^	0.53^b^	0.55^a^	**SQIw-MDS-N**
**<0.0001**	0.44^c^	0.49^b^	0.49^b^	0.53^ab^	0.55^a^	**SQIa-MDS-N**
**<0.0001**	0.31^c^	0.36^b^	0.37^b^	0.40^ab^	0.41^a^	**SQIn-MDS-N**

Note: same names than in Table 3. Similar letters indicate no significant difference at the 5% probability level.

Land uses with low-quality indexes require proper management practices to improve their SQI, ensuring that the properties affecting SQI move closer to their optimal range. This is especially important for MDS-selected characteristics, as they have the greatest impact on soil quality indexes.

Among all MDS properties, organic carbon showed the highest correlation with other soil properties. Managing organic carbon levels may help maintain other soil properties within their optimal ranges, thereby enhancing SQI in degraded land uses. Previous studies have demonstrated that without the addition of organic matter in the control treatment, a lower SQI was observed. The SQI was initially categorized in the 4^th^ class, but after the organic matter was added, its classification improved by 1 or 2 degrees [82]. Thus, controlling organic carbon content across different urban land uses is a key strategy for soil quality improvement.

Other indirect anthropogenic effects on soil formation factors, such as changes in carbon and nitrogen stocks, as well as soil temperature and moisture regimes [14], can either enhance or decrease the SQ depending on other conditions [83]. A comparison of the three indexes revealed that the Integrated and Weighted Integrated indexes had higher numerical values than the Nemoro index in both TDS and MDS, aligning with previous observations [33,84]. Regarding the linear approach applied to TDS indexes, the Weighted Integrated index exhibited the highest numerical values, whereas in MDS, the Integrated index had the highest numerical values. For the non-linear approach used in both TDS and MDS indexes, the Weighted Integrated and Integrated indexes displayed similar numerical values.

**Fig 4:**
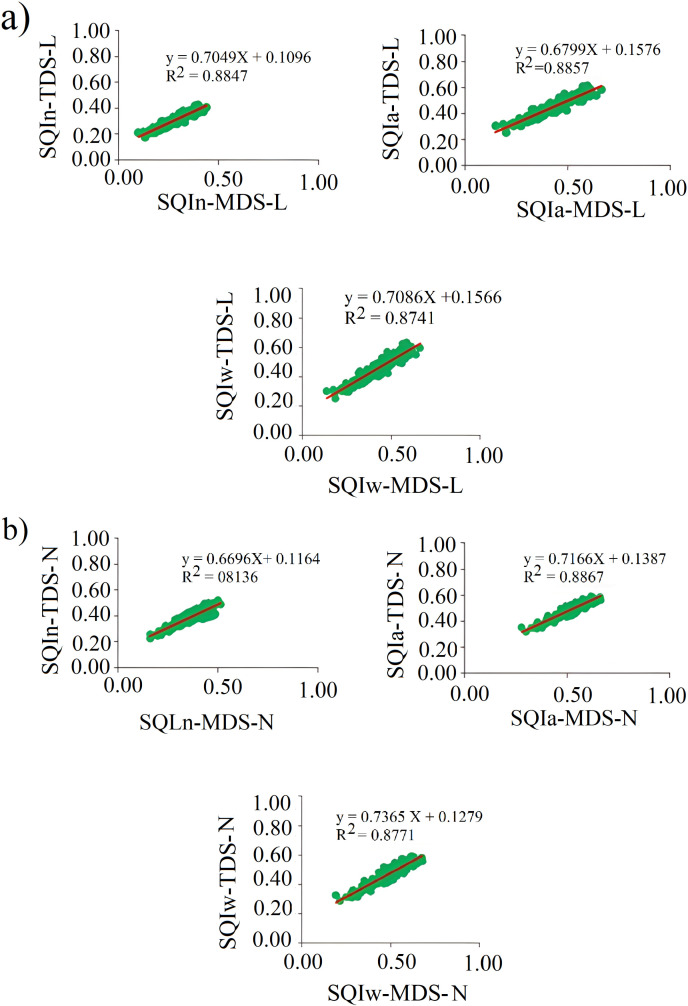
The linear relationship between TDS and MDS of soil quality indexes with (a) Linear calculation method (L) (b) Non-linear calculation method (N). Names following Table 3.

For the linear index calculation approach, the Integrated (SQIa) index had the highest R-squared value, while the Weighted Integrated (SQIw) index had the lowest R-squared value (Fig. 4a). In contrast, for the non-linear index calculation approach, the Integrated (SQIa) index again exhibited the highest R-squared value, whereas the Nemoro (SQIn) index had the lowest R-squared value (Fig. 4). These results indicate that SQI evaluation using MDS data—which requires analyzing fewer soil characteristics—can be performed with acceptable confidence, leading to time and cost savings.

Among the different methods, the Integrated SQI provided a more accurate assessment compared to Nemoro SQI. This is because, in addition to scoring, the Integrated index incorporates weighting for each soil property, whereas the Nemoro index only calculates mean values and considers the lowest-scoring property in its final assessment. Furthermore, the Nemoro SQI is less accurate because it does not account for the individual contributions of each property to overall soil quality. For instance, assuming equal importance for all soil properties prevents the model from reflecting the dominance of certain key factors in soil function. Additionally, the Nemoro index’s reliance on minimum values makes it highly sensitive to a single low-scoring property, which can disproportionately reduce the overall index, even when other properties perform well [85].

### 3.4. Soil quality map

**Fig 5:**
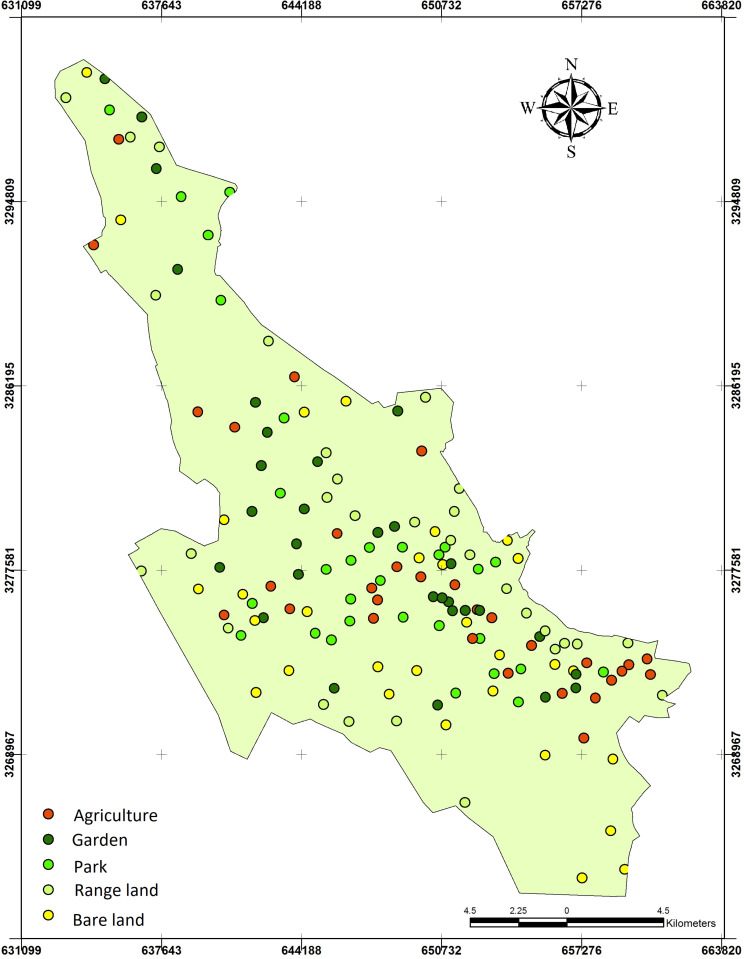
Land use sampling within the Shiraz urban watershed.

For a better understanding of SQI maps, it is essential to consider the land use variability within the study area (Fig 5). The urbanization intensity in Shiraz’s urban watershed is not uniform, leading to different land uses occurring over short distances. This variability is likely influenced by historical land use changes, including the conversion of certain areas into local parks to enhance green infrastructure. Such transformations contribute to the observed heterogeneous land use distribution across the region.

**Fig 6:**
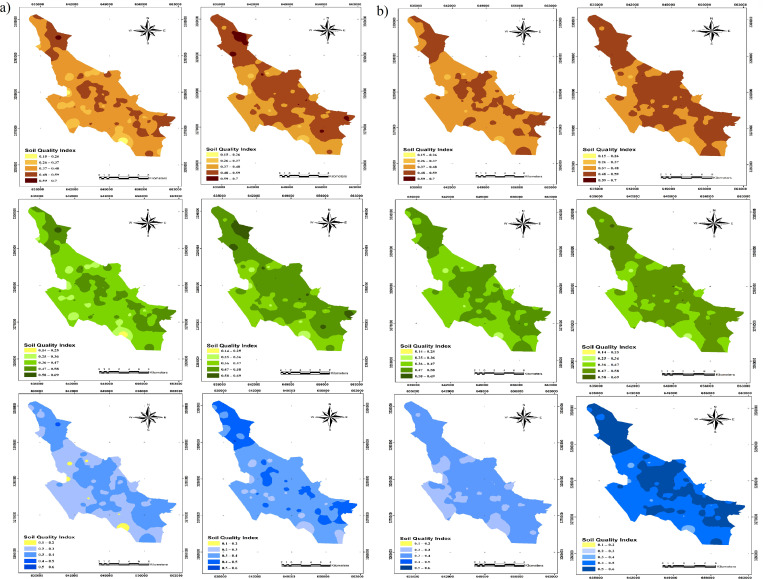
SQI maps classifying into five levels soil quality for all models, with lighter colors representing smaller values and darker colors indicating higher quality soils. In the “a” group on the left, the MDS models are shown, while in the “b” group on the right, the TDS models are presented. The Integrated method is represented by brown, the Weighted Integrated method by green, and the Nemoro method by blue. In each group, the linear approach is on the left, and the non-linear approach is on the right.

The SQI maps, calculated using both linear and non-linear approaches for TDS and MSD, are presented in Fig 6. By comparing Figs 5 and 6, it is evident that areas with concentrated land uses such as a parks or gardens exhibit higher soil quality.

This observation highlights the significant role of organic carbon and its annual return to the soil in enhancing soil quality. A general comparison between MDS and TDS maps suggests that MDS-based maps offer experts and decision-makers a reliable assessment while simultaneously reducing costs and time [84].

Urbanization and the associated anthropogenic activities significantly affect soil properties, often resulting in their degradation. These impacts alter various physical, chemical, and biological properties of soils, causing a decline in soil health and productivity. For example, the texture of soil, as described by Gee and Bauder [53], can be altered during urbanization due to the mixing or removal of natural soil horizons. Construction activities often replace native soils with materials of varying textures, leading to changes in soil water retention, aeration, and root penetration potential. Similarly, bulk density [55] increases substantially in urban soils due to compaction from heavy machinery, vehicular traffic, and pedestrian movement. Compacted soils exhibit reduced porosity, leading to impeded water infiltration, restricted root growth, and diminished aeration.

Furthermore, the stability of soil aggregates, often quantified by the mean weight diameter [56], is compromised in urban settings due to erosion, compaction, and the sealing of soil surfaces. These processes weaken aggregate cohesion, making soils more vulnerable to erosion and nutrient loss. Moreover, urban soils often exhibit a decline in available water [57] because of reduced organic matter content and increased sealing of soil surfaces with impervious materials like asphalt and concrete. The loss of porosity and organic matter reduces the soil’s water-holding capacity, leaving plants susceptible to drought. Moreover, Chemical properties of soils are equally affected in urban areas. Soil pH [59] is often altered due to the deposition of acidic pollutants from industrial emissions or alkalization from construction materials like lime and cement. Changes in pH disrupt nutrient availability and microbial activity, leading to a decline in soil fertility. Electrical conductivity and total dissolved salts [59,60] often increase in urban soils, driven by the application of saline irrigation water, road deicing salts, and excessive fertilizer use. This salinization process adversely affects plant growth and microbial diversity. The total neutralizing value [61] of urban soils declines when acidic inputs from industrial emissions and improper waste management overwhelm the soil’s buffering capacity. Furthermore, the loss of organic carbon [62] is a prominent feature in urban soils due to the removal of vegetative cover, reduced organic inputs, and the dominance of inorganic landscaping practices. This decline impacts soil structure [86], water retention, and biological activity. Alongside, cation exchange capacity [63] is diminished due to the erosion of organic matter and clay particles, reducing the soil’s ability to retain essential nutrients. Nutrient cycling in urban soils is disrupted, with notable declines in total nitrogen [64] and available phosphorus [65]. This is attributed to the lack of organic matter inputs and the fixation of phosphorus in unfavorable soil pH conditions. The availability of potassium [59] is also reduced due to leaching in compacted soils, further impairing plant growth and development. Urban soils are often contaminated with heavy metals such as lead, cadmium, arsenic, chromium, nickel, and cobalt [66] due to industrial emissions, vehicular exhaust, and improper disposal of hazardous materials. These contaminants accumulate in the soil, posing risks to plant, microbial, and human health. Iron content may also fluctuate depending on urban waterlogging conditions or changes in redox potential, which affect nutrient cycling and bioavailability. Finally, biological properties of soils are severely affected in urban areas. Microbial respiration [67] declines as soil compaction, contamination, and reduced organic inputs create unfavorable conditions for microbial communities. Similarly, the biomass of soil microbes, including carbon, phosphorus, and nitrogen [68], is significantly reduced, impacting nutrient cycling and overall soil health.

In conclusion, urbanization leads to profound and interconnected changes in soil properties. The physical, chemical, and biological degradation of soils in urban environments highlights the need for sustainable soil management practices. These may include the incorporation of organic amendments, reduction of compaction, mitigation of contamination, and restoration of vegetation to enhance soil quality and function.

## 4. Conclusion

The application of Integrated Quality Indexing (IQI) and Nemoro Quality Indexing (NQI) and their different scoring methods is not much common in urban and pre-urban areas. The current study aimed to investigate the capability of these methods in assessment of soil quality in such areas. The comparison of soil quality assessment methods related to land use in urban soils of Shiraz showed the Total Dataset (TDS) could be replaced by a Minimum Dataset (MDS), which effectively exhibited the quality of the studied soils. Additionally, organic carbon was placed as one of the soil properties in MDS, which had a significant effect on soil quality. This soil property was correlated with other properties and influenced them as well, helping to optimize soil quality. Different land uses represented different amounts of organic carbon resulting from the garden and bare land to show the highest and lowest SQI, respectively. Both Integrated and Weighted Integrated indexes showed higher numerical values than the Nemoro Index in both TDS and MDS. The SQI maps showed that replacing TDS with MDS could reduce costs and time when assessing soil quality. Considering that urbanization mainly causes the quality of soils to decrease, it is suggested to monitor the quality of urban soils continuously and to improve the quality of the soil by creating suitable urban green infrastructures to provide the food security and health of the beneficiaries of urban watersheds. Future research lines in metropolitan watersheds can be the application of satellite images, especially hyperspectral images in determining the quality of urban soils. This approach can decrease expenses and field sampling time, while improve region-wide soil quality monitoring. Soil-related indicators derived from satellite images such as soil moisture, organic carbon, vegetation health (NDVI), and erosion risks can be indirectly linked to soil quality and can improve the accuracy of assessments by integrating multiple quality indicators obtained from remote sensing. Furthermore, modern satellite sensors can provide detailed information about soil composition and texture, which could be combined with predictive modeling techniques such as machine learning. Also, information about climate variables, deforestation, and urbanization that affect soil quality can be obtained from satellite imagery to aid in having a holistic perspective in soil management.

## Supporting information

S1 File Supplementary data. (DOCX)
