# Supplementary material for: From fields to cities: Innovating assessment of soil quality in Southern Iran’s Urban areas
Source: PLoS One. 2025 May 9;20(5):e0321312. doi: 10.1371/journal.pone.0321312 (PMC12063871; doi:10.1371/journal.pone.0321312)
Supplement: S1 File — (DOCX) [file pone.0321312.s001.docx]

Table S1 in supplementary data is related to Table 7.

| **Table S1.** **Variance analysis of the impact of different land use on soil quality indicators** | | | | | | | | | | | | | | | |
| --- | --- | --- | --- | --- | --- | --- | --- | --- | --- | --- | --- | --- | --- | --- | --- |
| **Mean square** | | | | | | | | | | |  | | |  |  |
| **SQIw-TDS-L** | | **SQIa-TDS-L** | **SQIn-TDS-L** | **SQIw-TDS-N** | **SQIa-TDS-N** | **SQIn-TDS-N** | **SQIw-MDS-L** | **SQIa-MDS-L** | **SQIn-MDS-L** | **SQIw-MDS-N** | **SQIa-MDS-N** | | **SQIn-MDS-N** | **DF** |  |
| 0.053^**^ | | 0.042^**^ | 0.020^**^ | 0.049^**^ | 0.038^**^ | 0.033^**^ | 0.089^**^ | 0.083^**^ | 0.037^**^ | 0.068^**^ | 0.061^**^ | | 0.047^**^ | 4 | **Land use** |
| 0.005 | | 0.005 | 0.002 | 0.005 | 0.004 | 0.003 | 0.009 | 0.009 | 0.004 | 0.008 | 0.007 | | 0.007 | 145 | **Error** |
| Note: same names than in Table 3. * and ** are significant at the probability level of 1 and 5 percent, respectively. | | | | | | | | | | | | | | | |
